# A new genus and species of Mileewini leafhoppers (Hemiptera, Cicadellidae, Mileewinae) from China, with a key to genera

**DOI:** 10.3897/zookeys.1028.63727

**Published:** 2021-04-05

**Authors:** Bin Yan, Hong-Li He, Mao-Fa Yang, Mick D. Webb

**Affiliations:** 1 Institute of Entomology, Guizhou University; The Provincial Key Laboratory for Agricultural Pest Management of the Mountainous Region, Guiyang, Guizhou 550025, China; 2 College of Tobacco Science, Guizhou University; Guizhou Provincial Key Laboratory of Tobacco Quality Research, Guizhou University, Guiyang, Guizhou 550025, China; 3 Department of Life Sciences (Entomology), The Natural History Museum, London SW7 5BD, UK

**Keywords:** Auchenorrhyncha, Homoptera, identification, morphology, Old World, taxonomy

## Abstract

A new leafhopper genus and species, *Anzihelus bistriatus* Yan & Yang, **gen. nov. sp. nov.** (Cicadellidae, Mileewinae, Mileewini) is described from Sichuan Province, China. Habitus images and figures of the male and female genitalia are provided together with a key to the genera of Mileewini from China.

## Introduction

**Figure 1.**
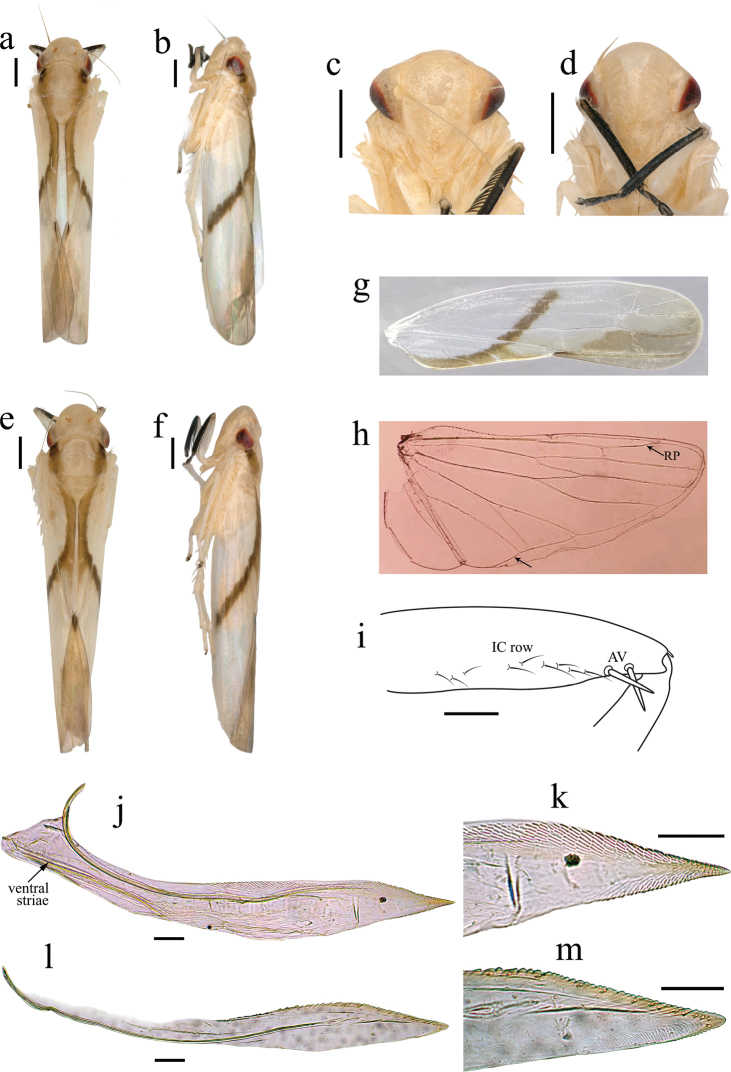
*Anzihelus bistriatus* sp. nov. **a–c** (male) **a** habitus, dorsal view **b** habitus, lateral view **c** face **d–f** (female) **d** face **e** habitus, dorsal view **f** habitus, lateral view **g–h** wings **g** forewing **h** hind wing **i** fore femur, anterior surface **j–m** valvulae **j, k** first valvulae, lateral view **l, m** second valvulae, lateral view (**l** damaged basally). Scale bars: 500μm (**a–f**); 0.1 mm (**i–m**).

**Figure 2.**
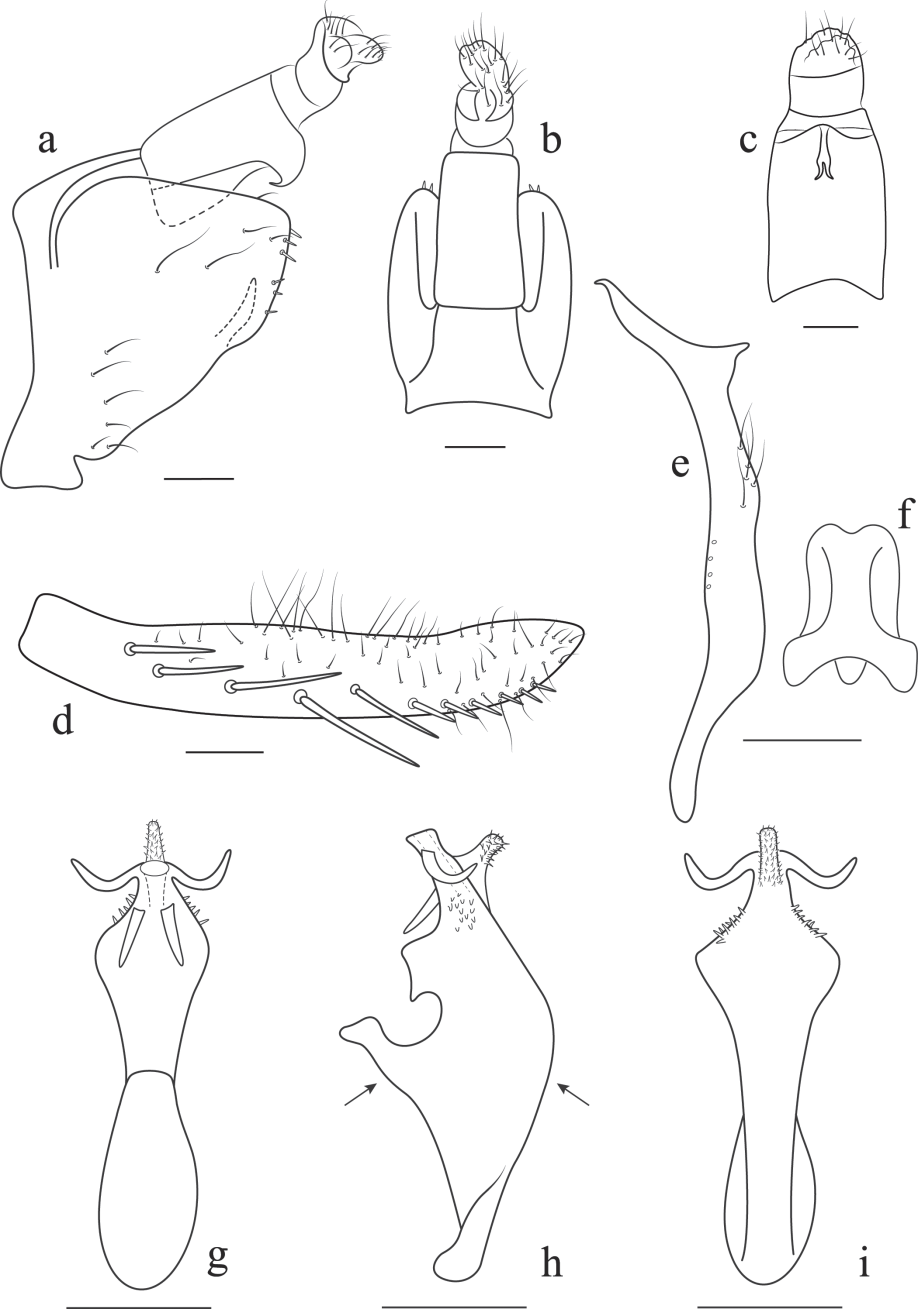
*Anzihelus bistriatus* sp. nov. **a, b** male genitalia **a** lateral view **b** dorsal view **c** anal tube, ventral view **d** subgenital plate, ventrolateral view **e** style, lateral view **f** connective, dorsal view **g–i** aedeagus **g** dorsal view (viewed from left-hand arrow in Fig. 2h) **h** lateral view **i** ventral view (viewed from right-hand arrow in Fig. 2h). Scale bars: 0.1 mm.

Mileewinae (Hemiptera, Cicadellidae) is a relatively small leafhopper subfamily comprising eight genera in four tribes distributed mainly in the Oriental and Ethiopian regions with a few species in the Neotropical and Palaearctic regions (Dietrich 2011; Krishnankutty and Dietrich 2011). At present, three genera with 69 species are recorded from China (He et al. 2021), all in the tribe Mileewini, a tribe distinguished in the Old World by the radial posterior hind wing vein (RP) extending to the anterior margin of the wing subapically (Fig. 1h) rather than apically. Due to the relatively small number of genera in the tribe it is of some interest that a new genus was discovered in China. In this article the new genus is described together with its type species and a key provided to the genera of Mileewini from China. The new genus is distinguished by its elevated head above the pronotum (Fig. 1b) and an unusual feature of the male Xth segment, i.e., with a single medial ventral process from its posterior margin (Fig. 2a, c); processes of the Xth segment are absent in other Mileewini but when present in other leafhoppers they are usually paired, caudally or anteriorly. Type specimens of the new species are deposited in the Institute of Entomology, Guizhou University, Guiyang, China (GUGC) and a single male paratype in the Natural History Museum, London, UK.

## Material and methods

The length of the body reported in the descriptions includes the forewings at rest. Morphological terminology follows Dietrich (2011). Male specimens were dissected under a Leica M125 microscope, then transferred to glycerine for further observation. Male genitalia were drawn using an Olympus CX41 microscope and drawings were enhanced using Adobe Illustrator CS6. Habitus photographs were taken with a Keyence VHX-6000 digital camera. All specimens studied are deposited in the Institute of Entomology, Guizhou University, Guiyang, China (**GUGC**).

## Taxonomy

### Key to the genera of Mileewini from China

**Table:** 

1	Forewing with apex truncate or emarginate and sometimes distinctly expanded from base to apex	*** Mileewa ***
–	Forewing with apex rounded, similar in width from base to apex (Fig. 1g)	**2**
2	Head equal in width to pronotum or slightly wider (Fig. 1a); male style apical process elongate, without setae at apex (Fig. 2e); female second valvulae evenly tapered to apex (Fig. 1l)	**3**
–	Head narrower than pronotum; male style apical process reduced with setae at apex; female second valvulae abruptly constricted subapically, beak-like (see He et al. 2018, fig. 33)	*** Processina ***
3	Head in profile level with pronotum, brown with yellow marking; male Xth segment (basal anal tube segment) without caudal process	*** Ujna ***
–	Head in profile elevated above level of pronotum (Fig. 1b), uniformly pale; male Xth segment (basal anal tube segment) with single caudal process (Fig. 2a, c)	***Anzihelus* gen. nov.**

#### Anzihelus gen. nov.

Taxon classificationAnimaliaHemipteraCicadellidae

D0204185-49FA-5AFE-9217-7F7822E7B62E

http://zoobank.org/37327FC6-BDF4-4516-A74F-5DDBDEF76292

##### Type species.

*Anzihelus bistriatus* sp. nov.

##### Description.

Small pale and slender leafhoppers. Head slightly wider than pronotum, shagreen; crown with anterior margin broadly rounded in dorsal view, median length almost equal to interocular width; shallowly concave at ocelli, the latter located slightly anterior to a line between anterior eye angles; laterofrontal sutures extending onto crown and attaining ocelli; coronal suture three-fifths median length. Face with frontoclypeus shallowly convex; anteclypeus slightly elevated in midline and slightly tapered to broadly rounded apex. Pronotum laterally carinate. Forewing with two open subapical cells; membrane equal in length to clavus. Hind wing with vein RP extending to anterior margin of wing subapically (Fig. 1h). Fore femur with two stout AV setae and several finer IC setae (Fig. 1i). Hindleg femoral setal formula 2:1:1.

***Male genitalia*.** Pygofer lobe in lateral view triangular with a weakly sclerotized band between lobe and dorsal bridge; ventroposterior margin with few spine-like setae; with short process arising at midlength of ventral margin, not attaining end of pygofer, apex pointed. Anal tube as long as pygofer, a medial lamellar process arising caudally from ventral surface, aligned vertically and bifurcate apically. Valve fused to pygofer. Subgenital plates long, narrow, apices exceeding posterior margin of pygofer; with uniseriate row of macrosetae from ventral margin in lateral view and some short and long microsetae on lateral surface. Connective Y-shaped, arms and stem short with a basomedial lobe. Style elongate apex foot-like with inner heel, with few fine setae on inner margin slightly distad of midlength. Aedeagus laterally compressed with distal processes on shaft, gonopore apical; basal apodeme short.

Female valvulae I in lateral view (Fig. 1j, k), with dorsal and ventral margins of shaft distinctly convex; apex acute; with diagonal strigate sculpture dorsally and apically; basal ventral striae present. Valvulae II in lateral view (Fig. 1l, m) leaf-shaped distally, apex subacute; blade dorsally with small and closely spaced crenulate sculpture and numerous irregular fine marginal teeth.

##### Remarks.

The new genus is similar to other Old World Mileewini genera (*Mileewa*, *Ujna* and *Processina*) in having vein RP in the hind wing extending to the anterior margin subapically (Fig. 1h) rather than the more usual apical margin and the marginal vein reaching the wing margin between Pcu and A1. It also has the basal ventral striae of the first valvulae found in the above genera (Fig. 1j). The latter feature, of unknown function, has been referred to as the ventral interlocking device but this function is performed by the rami of the first and second valvulae. The new genus can be distinguished from other Mileewini by its elevated vertex above the pronotum and from most other cicadellids by the male Xth segment (basal anal tube segment) with an unusual single caudal medial process (see also key to genera).

##### Etymology.

The genus takes its name from the locality of the type species, Anzihe Nature Reserve.

##### Distribution.

China (Sichuan).

#### Anzihelus bistriatus sp. nov.

Taxon classificationAnimaliaHemipteraCicadellidae

8F2F5225-E9DB-55B6-A877-0EC9588B80E0

http://zoobank.org/AE1ED0A1-00E8-4384-8113-63C44FF41BB2

Figs 1 , 2

##### Material examined.

***Holotype***: ♂, China, Sichuan Province, Chongzhou City, Anzihe Nature Reserve, 1595 m, 30 July 2016, coll. Bin Yan. ***Paratypes***: 6♂♂4♀♀, same data as holotype; 1♂, same data as holotype except, 1687 m, 31 July 2016, at light. All type species deposited in Institute of Entomology, Guizhou University, Guiyang, China (GUGC) and one male paratype in BMNH.

##### Description.

***Length*.** Male: 5.5–6.0 mm; female: 5.6–6.0 mm.

***Color*** pale yellow. Ocelli red or orange-yellow; eyes reddish brown laterally. Thorax with a brown band extending from behind each eye on pronotum, onto mesonotum and continued onto inner margin of clavus and diagonally across wing to costal margin. Forewing brownish hyaline distally. Legs with fore tibiae and tarsi black.

***Male genitalia*.** Genitalia as in generic description with aedeagus (Fig. 2g-i) broad in lateral view with shaft constricted and narrowed distally, with a short ventral process arising medially from ventral surface and a pair of short slightly more elongate processes arising subapically from dorsal surface and apically, all densely covered with micro-spines, lamellate medial lobe from dorsal surface basally.

***Female genitalia*.** Sternite VII, in ventral view, with length approximately equal to width; posterior margin convex medially.

##### Etymology.

The specific name refers to the two dark longitudinal stripes dorsally.

##### Remarks.

This new species can be easily recognized by its pale colour with a brown diagonal stripe across the forewings.

##### Distribution.

China (Sichuan).

## Supplementary Material

XML Treatment for Anzihelus

XML Treatment for Anzihelus bistriatus
